# From Neural Tube Formation Through the Differentiation of Spinal Cord Neurons: Ion Channels in Action During Neural Development

**DOI:** 10.3389/fnmol.2020.00062

**Published:** 2020-04-24

**Authors:** Raman Goyal, Kira A. Spencer, Laura N. Borodinsky

**Affiliations:** Department of Physiology & Membrane Biology and Institute for Pediatric Regenerative Medicine, Shriners Hospital for Children, University of California Davis School of Medicine, Sacramento, CA, United States

**Keywords:** neural tube formation, neural cell proliferation, neuronal differentiation, glutamate signaling, NMDA receptor, TRPM8, motor neuron differentiation, spinal cord development

## Abstract

Ion channels are expressed throughout nervous system development. The type and diversity of conductances and gating mechanisms vary at different developmental stages and with the progressive maturational status of neural cells. The variety of ion channels allows for distinct signaling mechanisms in developing neural cells that in turn regulate the needed cellular processes taking place during each developmental period. These include neural cell proliferation and neuronal differentiation, which are crucial for developmental events ranging from the earliest steps of morphogenesis of the neural tube through the establishment of neuronal circuits. Here, we compile studies assessing the ontogeny of ionic currents in the developing nervous system. We then review work demonstrating a role for ion channels in neural tube formation, to underscore the necessity of the signaling downstream ion channels even at the earliest stages of neural development. We discuss the function of ion channels in neural cell proliferation and neuronal differentiation and conclude with how the regulation of all these morphogenetic and cellular processes by electrical activity enables the appropriate development of the nervous system and the establishment of functional circuits adapted to respond to a changing environment.

## Introduction

Nervous system development is a complex process in which neural cells undergo a transformation from neural stem cells to highly specialized neurons and glia to form different brain structures and spinal cord and establish circuitry that facilitates simple to advanced neural functions.

Many cues have been recognized as drivers of the first steps in nervous system development. Morphogenetic proteins and growth factors regulate the number and type of neural cells as well as the morphogenesis of the neural tube. These include Sonic hedgehog (Shh), Bone Morphogenetic Proteins (BMPs), Wnts and trophic factors such as EGF, IGF, NGF, BDNF to mention few. Most of these factors are not exclusive to the organogenesis of the brain and spinal cord but instead support growth and act as morphogens of every tissue and organ in the developing embryo. Mechanistically, these developmental cues trigger a transcriptional combinatorial code that accompanies cells through their cell cycle progression and exit, differentiation and maturation (Cohen et al., 2013; Bier and De Robertis, 2015).

Also, some of the pathways triggered by these proteins intertwine with second messenger signaling, like those driven by spatiotemporal changes in cAMP, inositol triphosphate (IP3) and Ca^2+^ concentrations, through the recruitment of specialized enzymes that imprint posttranslational modifications in effector proteins (Borodinsky et al., 2015). For instance, in embryonic *Xenopus* spinal cord opposing gradients of BMPs and Shh regulate neuronal differentiation across the dorsoventral axis by modulating the frequency of Ca^2+^ transients in developing neurons (Belgacem and Borodinsky, 2011; Swapna and Borodinsky, 2012). While Shh increases Ca^2+^ spike activity through recruiting transient receptor potential channels (TRPC) and IP3 receptor-operated Ca^2+^ release from stores in ventral domains of the spinal cord (Belgacem and Borodinsky, 2011, 2015), BMPs decrease Ca^2+^ spike activity of dorsal neurons through the activation of p38 MAP kinase and inhibition of Na^+^ conductance necessary for activating voltage-gated Ca^2+^ channels (Swapna and Borodinsky, 2012). Similarly, morphogenetic proteins of the Wnt family acting through non-canonical pathways (Slusarski et al., 1997; Sheldahl et al., 1999) regulate neuromorphogenesis. Specifically, Wnt5a recruits the receptors Frizzled and Ryk that trigger Ca^2+^ transients mediated by TRPC and IP3 receptors to regulate axon growth and guidance of rodent corticospinal neurons grown *in vitro* (Li et al., 2009). All these studies share a common effector that is Ca^2+^ dynamics. This indicates that neural activity, a modifier of [Ca^2+^]_i_, might also be a driving force for neural development either in concert or independently of morphogenetic protein actions.

Neural activity is a feature of the maturing and mature nervous system, which during development facilitates the refinement of neural connections. The expression of ion channels in mature neurons is intrinsic to neuronal function. Diverse ion conductances are indispensable for neurotransmission, thus, the roles of different ion channels in synaptic function and neuronal excitability have been extensively studied. In contrast, the neurophysiological features of neural cells before synapse formation and before neuronal differentiation has not been as strong a focus of attention as those of mature neurons. Nevertheless, studies have argued that other forms of neural activity are present in neural cells throughout nervous system development (Spitzer, 2006; Smith and Walsh, 2020).

This activity may not be structured under a classical chemical synapse, but it is certainly dependent on ion channels gated by diverse mechanisms. Expression of voltage- and neurotransmitter-gated ion channels as well as transient receptor potential (TRP) channels, among others, is apparent in neural stem cells as early as neural plate stages (Abdul-Wajid et al., 2015; Sequerra et al., 2018; Spencer et al., 2019). Moreover, ion channels have been shown to participate in the formation of the brain and spinal cord during one of the first developmental steps known as neural tube formation (Abdul-Wajid et al., 2015; Sequerra et al., 2018).

Here, we review studies addressing the pattern of expression of ion channels during development in neural cells before and during synapse formation. We compile investigations demonstrating a role for ion channels in neural cell proliferation, neural tube formation, and neuronal differentiation and discuss the consequences of having neural activity functioning in the early stages of nervous system development.

## Ontogeny of Ion Channel Expression in Excitable Tissues

The excitable nature of neurons and muscle cells is dependent on the specific expression of ion channels and their subcellular localization in these cells. Seminal studies have investigated the developmental appearance of excitability in neurons and muscle cells through the progressive and differential expression of ion channels. Embryonic spinal cord neurons have served as a powerful model for the study of the ontogeny of excitability during development. Action potentials in *Xenopus laevis* spinal cord neurons are first recorded 8 h after exiting the cell cycle, when, these events manifest spontaneously, are Ca^2+^-dependent and long in duration (Spitzer and Lamborghini, 1976; Holliday and Spitzer, 1990; Gu et al., 1994; Gu and Spitzer, 1995). Developmental upregulation in the expression of an inward rectifier voltage-gated K^+^ channel contributes to shorten the action potential duration and shifts it from Ca^2+^- to Na^+^-mediated (Barish, 1986; O’Dowd et al., 1988). The identity of specific Ca^2+^, Na^+^ and K^+^ voltage-gated channel subunits for which their expression is developmentally regulated have been investigated (Harris, 1988; Ribera and Spitzer, 1992; Spitzer and Ribera, 1998). In particular, Kv1.1 and Kv2.2 appear progressively and respectively in immature and mature spinal cord neurons to contribute to the increased K^+^ current as development advances (Gurantz et al., 1996). Similarly, studies in other species have shown developmentally-regulated expression of ion channels during spinal cord neuron differentiation that results in the progressive appearance of ionic currents in these neurons. For instance, T-type Ca^2+^ currents are dominant at the earliest embryonic stage of chick limb motor neuron development, while later T currents decrease and N and L Ca^2+^ currents increase (McCobb et al., 1989). Moreover, changes in Na^+^ and K^+^ currents in these motor neurons during embryonic development result in changes in action potential amplitude and duration, respectively, which in turn, modify the instructions of motor neurons to the muscle (McCobb et al., 1990).

In addition to voltage-gated ion channels and their fundamental role in contributing to the excitability of developing neurons and muscle cells, other types of ion channels are also present at the early stages of embryonic development. These channels are gated by diverse mechanisms, including notably, neurotransmitter-operated channels. GABA and glutamate receptors are expressed in immature *Xenopus* spinal cord neurons and their activation contributes to the spontaneous Ca^2+^ spike activity in these cells before and during synapse formation (Root et al., 2008). Glutamate-operated channels are involved in the electrical coupling of developing mouse motor neurons (Personius et al., 2008). Expression of subunits of acetylcholine-gated channels, is developmentally regulated in chicken motor neurons and skeletal muscle (Keiger et al., 2003). Several subunits are expressed in motor neurons and muscle before muscle innervation and others are downregulated after completion of apoptosis of developing motor neurons (Keiger et al., 2003). Similarly, NMDA receptors are present and active at the neuromuscular junction during motor neuron axon pruning in early postnatal mouse development (Personius et al., 2016).

Our recently published study shows that the cold-sensitive channel TRPM8 is expressed in the developing *Xenopus* embryo (Spencer et al., 2019). Both mRNA and protein are detected since the early stages of neural tube formation (Spencer et al., 2019) and transcripts are enriched in neural tissue (Session et al., 2016). During spinal neuron differentiation, TRPM8 protein appears enriched in the ventral domain of the embryonic spinal cord and makes a major contribution to the calcium spike activity of ventral spinal cord neurons at cold temperatures (Spencer et al., 2019). Similarly, in other species developing spinal cord neurons also express temperature-sensitive ion channels including motor neurons, which express TRPV2 that regulates axon outgrowth (Shibasaki et al., 2010), and in early postnatal mouse motor neurons contributes to their electrical properties (Bouhadfane et al., 2013).

Expression of these diverse types of channels appears to start at even earlier stages of neural development, before the neural tube is formed. At neural plate stages, *Xenopus* neuroectodermal cells exhibit Ca^2+^ transients (Abdul-Wajid et al., 2015; Christodoulou and Skourides, 2015; Sequerra et al., 2018) that are mediated partially by T-type Ca^2+^ channels (Abdul-Wajid et al., 2015) and by NMDA receptors, as demonstrated by our recently published study (Sequerra et al., 2018).

Transcripts and proteins for glutamate (Root et al., 2008; Session et al., 2016; Sequerra et al., 2018) and GABA (Barker et al., 1998; Root et al., 2008; Session et al., 2016) release and reception, among many other neurotransmitters (Choi et al., 1998; Messenger et al., 1999), are detected during neural plate stages. Accordingly, the role of neurotransmitter signaling and ion channels in neural tube formation demands further investigation.

## Neural Tube Formation and Ion Channels

The process of neural tube formation consists of transforming a flat layer of cells known as the neural plate into a tubular structure from which the brain and spinal cord originate. The cellular events encompassing neural tube morphogenesis, all of which are tightly regulated, include neural plate cell proliferation, apicobasal polarization, apical constriction, elongation, cell intercalation, migration and differentiation (Wallingford et al., 2013; Nikolopoulou et al., 2017). Intriguingly, the use of antiepileptic drugs (AEDs) during pregnancy increases the incidence of neural tube defects (NTDs) by unclear mechanisms (Robert and Guibaud, 1982; Lindhout and Schmidt, 1986; Rosa, 1991).

**Figure 1:**
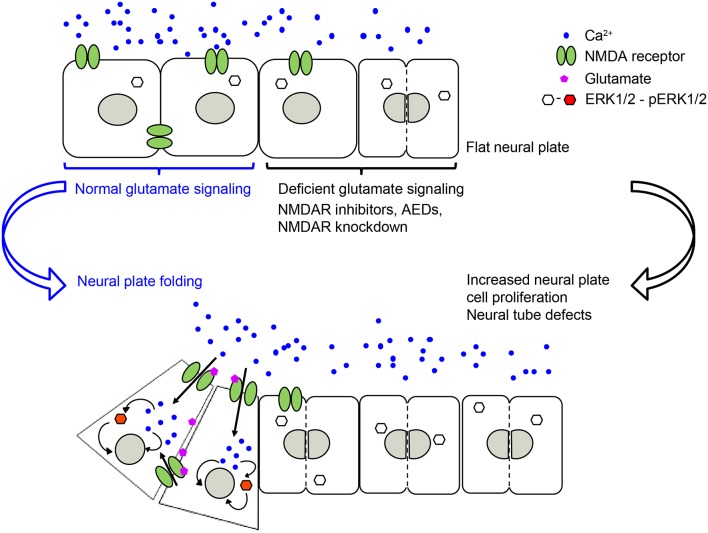
NMDA receptor-mediated signaling is necessary for regulating neural plate cell proliferation in neurulating *Xenopus laevis* embryos, which in turn is required for appropriate morphogenesis of the neural tube. Genetic or pharmacological inhibition of NMDA receptor activity, including antiepileptic drugs (AEDs) leads to an increase in proliferating neural plate cells, which in turn leads to neural tube defects (NTDs). Based on Sequerra et al. (2018).

Our recently published study (Sequerra et al., 2018) shows that glutamate signaling is present during neural plate stages in *Xenopus laevis* embryos. We demonstrated that during neural tube formation neural plate cells exhibit Ca^2+^ transients partly mediated by NMDA receptors. Inhibiting glutamate signaling, through pharmacological inhibition of NMDA receptors or downregulation of the GluN1 subunit, induces NTDs (Figure 1). Valproic acid, an AED known to increase the incidence of NTDs in humans and animal models (Rosa, 1991; Lindhout et al., 1992; Padmanabhan and Ahmed, 1996), also inhibits Ca^2+^ dynamics in the neural plate to a similar extent as inhibition of NMDA receptors. Moreover, preincubating embryos with NMDA partially rescues both the number of Ca^2+^ transients in the folding neural plate and the valproic acid-induced NTD phenotype (Sequerra et al., 2018). Additionally, both valproic acid- and deficient NMDA receptor signaling-induced NTDs are completely rescued by enhancing ERK1/2 activation (Sequerra et al., 2018). These findings demonstrate that neurotransmitter signaling is present during the earliest stages of nervous system development and is fundamental for the morphogenesis of the neural tube (Figure 1). These discoveries suggest that primary targets of AEDs are already present and functional in neural plate stages. Thus, exposure of the fetus to these drugs during the critical period of neural tube formation may interfere with necessary neural activity and signaling leading to NTDs.

Many other neurotransmitter signaling systems have been identified as participants in the process of neural tube formation. Inhibiting serotonin receptors 5HT_2B_ interferes with mouse neural tube closure and morphogenesis (Choi et al., 1998). The expression of these receptors during neural tube formation appears enriched in neural crest cells, which also explains morphological defects of the developing heart (Choi et al., 1998).

Administering GABA_A_ and GABA_B_ receptor ligands to pregnant rats alters embryos’ neural tube formation leading to NTDs (Briner, 2001). The fact that both agonists and antagonists of GABA receptors elicit these defects suggest that balanced signaling is required for the appropriate morphogenesis of the neural tube. Additionally, incubating elevated-neural-fold stage rat embryos with diazepam for 24 h prevents neural tube closure (Smedley and Stanisstreet, 1986), which may result from targeting the benzodiazepine domain of GABA_A_ receptors. Another chloride conductance-mediated neurotransmitter system that affects neural tube formation is glycine. Blocking glycinergic signaling by administering strychnine to pregnant rats results in embryos with anencephaly, anterior NTD (García-Alcocer et al., 2005).

Similarly, enhancing or inhibiting NO levels by enhancing BMP signaling or inhibiting NO synthase in chicken embryos induces NTDs (Traister et al., 2004). Low NO levels appear to facilitate neural plate cell proliferation and to decrease apoptosis, and vice-versa when NO levels are high. Hence, NO signaling dynamically regulates the number of neural plate cells that in turn is important for neural tube morphogenesis (Traister et al., 2004).

Noradrenaline promotes neuronal differentiation by upregulating expression of N-tubulin in noggin-expressing neural plate cells, which is prevented by inhibiting α-adrenergic receptors (Messenger et al., 1999). Noradrenergic signaling in the neuroectoderm promotes further neuronal differentiation by enabling the expression of dedicated neurofilament-associated protein related to the acquisition of specialized neuronal morphology and function (Messenger et al., 1999).

Ca^2+^ signaling is a plausible common denominator for the action of diverse neurotransmitter systems on neural tube formation. Sources of Ca^2+^ can be intracellular from stores or extracellular through Ca^2+^ influx. Early studies in cultured rat embryos during cephalic neural fold elevation and neural tube closure assessed the role of Ca^2+^ influx and found that reducing it causes opening of the elevated neural folds (Smedley and Stanisstreet, 1986). We found that inhibiting the NMDA receptor function decreases the number of Ca^2+^ transients in the neural plate, suggesting that additional mechanisms other than these ionotropic glutamate receptors contribute to neural plate cell Ca^2+^ dynamics during folding (Sequerra et al., 2018). An interesting pattern of ionic current is present in the folding neural plate in *Xenopus laevis* embryos consisting of a Na^+^-dependent inward current which is stronger in the mid-lateral neural plate and decreases near the midline of the neural groove (Robinson and Stump, 1984). Similarly, a comparable mediolateral pattern of resting membrane potential is observed during neural plate folding in this species (Pai et al., 2015). Disruption of this membrane potential pattern, by overexpression of the potassium channel Kv1.5 or GlyR and incubation with chloride channel agonist, leads to defects in brain morphogenesis (Pai et al., 2015). The level of membrane polarization, the makeup of ion channels expressed in the neural plate and the exposure to mediators or modulators of neurotransmitter signaling will differentially recruit Ca^2+^ currents with characteristic kinetic parameters to transduce the signaling required for neural tube morphogenesis. Indeed, T-type Ca^2+^ channels are involved in neural tube formation and loss of them impairs neural fold closure in *Xenopus laevis* and *Ciona* embryos. Ca^2+^ influx through these channels is necessary for the regulation of cell adhesion during neural tube formation by ephrin signaling (Abdul-Wajid et al., 2015). Furthermore, these Ca^2+^ transients seem to regulate apical actin dynamics in superficial neural plate cells of *Xenopus laevis* embryos, which, in turn, regulates neural plate cell apical constriction necessary for neural tube formation (Christodoulou and Skourides, 2015).

Further investigation is needed to identify the molecular mechanisms eliciting Ca^2+^ signaling and downstream effectors recruited for neural plate folding and neural tube formation. The elucidation of these mechanisms will contribute to the delineation of safe therapies for the treatment of epilepsy during pregnancy.

## Neural Cell Proliferation and Ion Channels

The generation of the appropriate number of neurons and glial cells is essential not only during nervous system development but also in the adult brain where neurogenesis occurs in the hippocampus and olfactory bulb, and the peripheral nervous system during regeneration and remodeling. Thus, this is a highly regulated process because the dysregulated proliferation of neural stem cells can lead from tumors to neurodevelopmental disorders and birth defects like NTDs.

The expression of ion channels during the early stages of development supports a role for them in the relevant cellular processes pertinent to these stages including neural cell proliferation. Different types of ion channels including voltage-gated, neurotransmitter-gated, TRPC and store-operated Ca^2+^ channels have all been implicated in regulating neural plate cell proliferation.

The action of glutamate-mediated regulation of neural plate cell proliferation is apparent as early as neural plate stages. We found that blocking NMDA receptor signaling increases neural plate cell proliferation in *Xenopus* embryos, and, likely as a consequence, impairs lateromedial migration leading to NTDs. An increase in neural plate cell proliferation is also apparent by incubating embryos with the AED valproic acid (Sequerra et al., 2018), in contrast to the off-target inhibitory effect of valproic acid on cell proliferation as an inhibitor of histone deacetylase (Lane and Chabner, 2009). Altogether, these studies suggest that it is the AED action on its primary targets and its interference with neural activity that is responsible for inducing NTDs due to enhancing neural plate cell proliferation.

Regulation of neural plate cell proliferation by glutamate-gated ion channels is present at later developmental stages during corticogenesis in the rodent brain. The effects of glutamate signaling on neural progenitor proliferation vary depending on the nervous system structure, the developmental stage and the type of model system and manipulation used in specific studies. Glutamate decreases the number of proliferating embryonic rat cortical cells through an AMPA/Kainate receptor-dependent mechanism that leads to depolarization of neural progenitors in the ventricular zone and activation of voltage-gated Ca^2+^ channels (LoTurco et al., 1995). In contrast, during embryonic rat striatal neurogenesis, it has been shown that glutamate signaling increases neural progenitor proliferation by an NMDA receptor-dependent mechanism in the ventral telencephalon (Luk et al., 2003). *In vitro* studies on embryonic rat hippocampal neural progenitors reveal that stimulating glutamate signaling enhances neural cell proliferation or neuronal differentiation depending on the temporal pattern of NMDA receptor activation (Joo et al., 2007), suggesting that the apparent discrepancies between studies focused on different brain regions, developmental stages and experimental preparations might be rooted in different spatiotemporal profiles of downstream glutamate signaling.

Another important neurotransmitter-gated ion channel that participates in neural progenitor cell proliferation is the GABA_A_ receptor. *In vivo* studies in the neonatal mouse subventricular zone (Young et al., 2012) and *in vitro* studies in cerebellar granule cells (Fiszman et al., 1999) show that GABA_A_ receptor-induced depolarization enhances neural progenitor cell proliferation. In contrast, activation of this receptor in the ventricular zone of the rat embryonic neocortex (LoTurco et al., 1995) and isolated neural precursor cells of the early postnatal rat striatum (Nguyen et al., 2003) show that GABA signaling recruits voltage-gated Ca^2+^ channels and inhibits cell cycle progression. Even in the adult mouse brain, the GABA_A_ receptor seems crucial in regulating neurogenesis by controlling hippocampal neural progenitors. GABA_A_ receptor γ2 subunit-mediated signaling is responsible for controlling the experience-dependent transition between quiescence vs. proliferative states of the mouse hippocampal neural stem cell niche (Song et al., 2012). In particular, γ2-GABA_A_ receptor favors the quiescence of adult neural stem cells (Song et al., 2012). Moreover, a diazepam binding inhibitor is expressed in neural stem cells of the postnatal hippocampal subgranular zone and enhances proliferation by dampening GABA_A_ receptor signaling (Dumitru et al., 2017). All of these studies clearly demonstrate a role for glutamate- and GABA-gated ion channels in regulating neural progenitor proliferation.

Other ion channels directly involved in regulating neural cell proliferation include the voltage-gated ion channels. Moreover, some actions of the neurotransmitter receptor-gated ion channels seem to converge into the recruitment of voltage-gated ion channels *via* membrane depolarization. In particular, voltage-gated Ca^2+^ channels are pivotal for the regulation of neural progenitor proliferation and mouse embryonic cortical layer formation (Malmersjö et al., 2013). Voltage-gated Ca^2+^ channels enable Ca^2+^ transients that propagate through a network of neural progenitors connected by gap junctions. Inhibiting this electrotonic transmission decreases neural progenitor proliferation suggesting that correlated Ca^2+^ transients are necessary for the regulation of neural progenitor proliferation (Malmersjö et al., 2013). In contrast, in frog embryos membrane hyperpolarization that presumably impedes activation of voltage-gated Ca^2+^ channels in embryonic neural cells increases neural cell proliferation that has detrimental consequences to brain morphogenesis (Pai et al., 2015). These studies suggest that as the differential impact glutamate signaling has on neural proliferation and differentiation, the impact voltage-gated Ca^2+^ channel-mediated signaling has, depends on developmental timing and location.

In addition to Ca^2+^ channels, voltage-gated K^+^ and Na^+^ channels are involved in regulating neural cell proliferation. The voltage-gated Na^+^ channel β1 subunit is necessary for inhibiting granule cell precursor proliferation during the first week of mouse postnatal dentate gyrus development (Brackenbury et al., 2013). Another instance of the role of voltage-gated Na^+^ channels was discovered recently in *Drosophila* larvae, where the single pore-forming voltage-gated Na^+^ channel α subunit, paralytic, regulates neural progenitor proliferation and survival (Piggott et al., 2019). In the adult nervous system the epithelial Na^+^ channel regulates neural stem cell proliferation in the subependymal zone and consequently neurogenesis in the mouse olfactory bulb (Petrik et al., 2018). In zebrafish, the homolog of voltage-gated K^+^ channel α-subunit K_v_6.4 regulates the proliferation of cells lining the embryonic brain ventricles (Shen et al., 2016). A gain-of-function mutation of this subunit decreases cell proliferation, while the loss of K_v_6.4 increases it. Moreover, the K_v_6.4 action appears antagonized by the expression of a homolog to the delayed rectifier K^+^ channel subunit K_v_2.1, for which gain and loss of function manipulations cause the opposite effects on neural progenitor proliferation and ventricular brain development (Shen et al., 2016).

Non-voltage-gated ion channels have also been implicated in regulating neural cell proliferation. For instance, TRPC1 participates in bFGF/FGFR1-mediated proliferation of embryonic rat neural stem cells through a Ca^2+^-dependent mechanism (Fiorio Pla et al., 2005). Similarly, TRPC1 mediates the proliferative effect of PDGF-BB on rat hippocampal neuronal progenitors, which rescues the detrimental effect on neurogenesis inflicted by HIV infection and cocaine (Yao et al., 2012). Embryonic and adult mouse neural stem cells express Ca^2+^ release-activated Ca^2+^ channels that enable store-operated Ca^2+^ entry in these cells mediated by Orai1 and STIM1. Downregulating the expression of these molecules decreases *in vitro* and *in vivo* proliferation of neural stem cells (Somasundaram et al., 2014).

The specific effect that is triggered downstream of all these ion channels on cell proliferation varies among these studies. This is likely due to differences in downstream signaling, recruitment of molecular partners, environmental milieu and maturational status of the cells subjected to these ion channel-triggered signaling mechanisms. There are many downstream signaling effectors reported to mediate ion channel-dependent regulation of neural cell proliferation. For instance, downstream of voltage-gated K^+^ channels and membrane depolarization, oligodendrocyte progenitor proliferation is regulated by controlling the progression of the cell cycle at the G1 phase, likely through a cAMP and cyclin-dependent kinase inhibitors p27Kip1 and p21CIP1 mechanism (Ghiani et al., 1999). In contrast, downstream of GABA_A_ receptor-triggered hyperpolarization in embryonic and neural crest stem cells, S-phase checkpoint kinases of the phosphatidylinositol-3-OH kinase-related kinase family and, subsequently the histone variant H2AX, regulate proliferation (Andäng et al., 2008). Recruitment of the ERK pathway is a shared effector of several ion channel-regulated neural cell proliferation pathways. TRPC1 activation is necessary for the recruitment of PDGF-BB-induced ERK/CREB and mTOR/eukaryotic translation initiation factor 4E-binding protein-p70S6K and nuclear factor-κB in rat hippocampal neural progenitors (Yao et al., 2012). Similarly, activation of the epithelial Na^+^ channel by fluid flow elicits Ca^2+^ dynamics through Ca^2+^ release-activated channels that activate ERK, regulating neural cell proliferation in the mouse subependymal zone in the olfactory bulb (Petrik et al., 2018). Additionally, stimulation of the NMDA receptor *in vivo* activates ERK in neural plate cells during *Xenopus* neural tube formation. In turn, constitutive activation of ERK during neural plate folding completely rescues the NTD phenotype induced by dysregulated neural plate cell proliferation due to blocking NMDA receptors or incubation with the AED valproic acid (Sequerra et al., 2018; Figure 1).

## Neuronal Differentiation and Ion Channels

It has been long recognized that expression of ion channels and prominently voltage-gated ion channels, their density, clustering, and subcellular localization are at the core of what distinguishes a neuron from other cell types. This fundamental question was addressed by pioneering studies from the Mandel lab when they cloned the transcription factor REST and identified it as a silencer element active in nonneuronal cells. In contrast, the absence of REST in neurons allows for the expression of the type II Na^+^ channel, which in turn assigns a functional neuronal identity to developing nervous system cells (Maue et al., 1990; Kraner et al., 1992; Chong et al., 1995). In turn, the specific makeup of ion channels expressed by developing neurons is essential for the different aspects of neuronal differentiation, including the acquisition of appropriate morphology, specification of neurotransmitter phenotype, axonal and dendritic growth and pathfinding, synaptogenesis and establishment of connections with corresponding partners, and physiological features that contribute to the precise characteristics of the developing circuit in which each differentiating neuron is meant to participate. Work from the Spitzer lab identified that Ca^2+^ spikes generated by voltage-gated Ca^2+^ and Na^+^ channels in embryonic neurons (Gu et al., 1994) regulate the specification of neurotransmitter phenotype that spinal cord mature neurons exhibit at larval stages of *Xenopus laevis* development (Gu and Spitzer, 1995; Borodinsky et al., 2004). These studies demonstrated that the frequency of Ca^2+^ spikes that embryonic neurons exhibit is important for the type of neurotransmitter spinal cord neurons express, following a homeostatic rule by which higher spike frequency leads to expression of inhibitory neurotransmitters while suppression of these spikes results in expression of excitatory neurotransmitters (Borodinsky et al., 2004). They also found that Ca^2+^ waves, dependent on both extracellular Ca^2+^ and Ca^2+^ release from ion channels present in intracellular Ca^2+^ stores such as ryanodine receptors, are apparent in growth cones of developing neurons (Gu et al., 1994) and are important for axon extension and guidance (Gu et al., 1994; Gu and Spitzer, 1995; Gomez and Spitzer, 1999).

K^+^ channels have been at the center of the process of neuronal differentiation in a variety of central nervous system structures and species. Weaver mice carry a mutation in a G-protein coupled inward rectifier K^+^ channel, GIRK2, that affects the pore-forming domain of the protein and impairs cerebellar granule neuron differentiation immediately after cell cycle exit (Patil et al., 1995). Expression of K^+^ channels, in turn, is a key determinant of the spontaneous Ca^2+^ activity that developing neurons exhibit. For instance, K^+^ channels like the small conductance Ca^2+^-activated K^+^ channel SK2 are expressed in immature Purkinje cells in the postnatal rat cerebellum and establish a feedback loop with Ca^2+^ influx and activation of Ca^2+^ channels to regulate the spatiotemporal features of Ca^2+^ transients in developing neurons (Cingolani et al., 2002). In particular, the developmentally regulated expression of SK2 elicits an after hyperpolarizing current in immature Purkinje neurons regulating their firing pattern during neuronal differentiation (Patil et al., 1995). Similarly, the acquisition of a mature neuronal action potential during spinal cord neuron differentiation is dependent on the expression of a delayed rectifier K^+^ channel during a critical period of development (Ribera and Spitzer, 1989, 1992).

**Figure 2:**
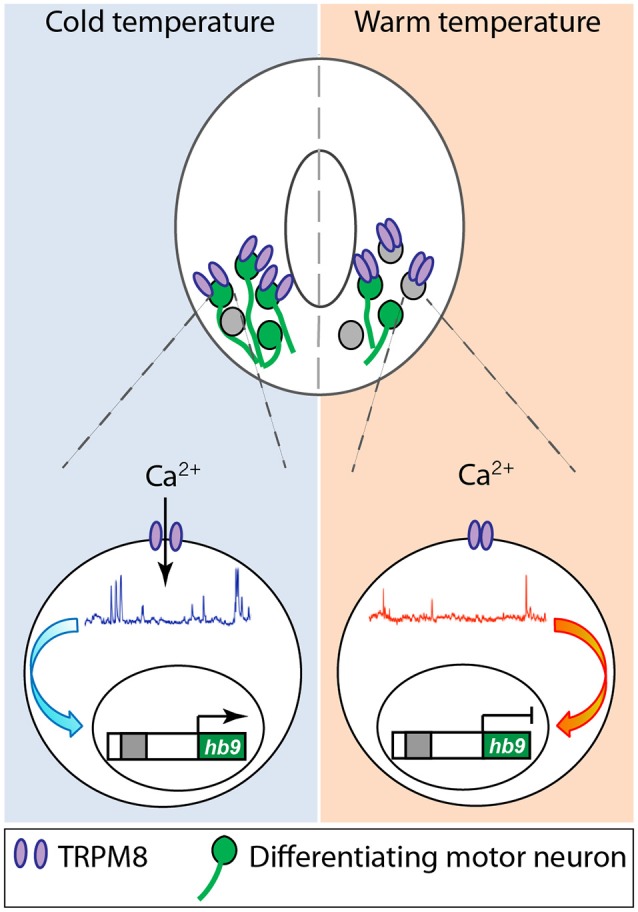
Cold temperature activates TRPM8 expressed in embryonic *Xenopus laevis* ventral spinal cord neurons, increasing Ca^2+^ spike frequency that upregulates expression of the motor neuron phenotype master transcription factor hb9, resulting in an increase in number of motor neurons in animals grown at cold temperature. Based on Spencer et al. (2019).

While all these studies are focused on specific ion channels and their role in different aspects of neuronal differentiation, they converge on shaping the spontaneous Ca^2+^ activity that differentiating neurons exhibit during development either directly or indirectly. Another instance of direct regulation of Ca^2+^ activity in developing neurons that connects the process of differentiation with the environment in which embryos develop is represented by the role of TRPM8, cold-sensitive channel, in spinal cord neuron differentiation (Spencer et al., 2019; Figure 2). Activation of TRPM8 at cold temperature increases Ca^2+^ spike frequency in ventral spinal cord neurons of *Xenopus laevis* embryos. In turn, Ca^2+^ spike activity regulates the expression of Hb9 (Spencer et al., 2019), a transcription factor known to be necessary for motor neuron phenotype specification and maintenance (Arber et al., 1999; Thaler et al., 1999). As a result, embryos grown at cold temperatures develop into larvae that possess a higher number of differentiated motor neurons innervating the axial musculature (Spencer et al., 2019; Figure 2). Similarly, mushroom body Kenyon neurons from Drosophila flies raised in high temperatures exhibit an increase in high-order axonal branching that is dependent on temperature-dependent excitability of these developing neurons mediated by Na^+^, Ca^2+^, and K^+^ channels and the downstream interaction between Ca^2+^ and cAMP dynamics (Peng et al., 2007).

Neurotransmitter-gated channels that regulate neuronal differentiation also involve Ca^2+^ dynamics downstream of neurotransmitter receptor activation. NMDA receptor function is necessary for the development of dendritic arbors in differentiating retinotectal neurons and to establish appropriate retinotectal topographic maps (Cline and Constantine-Paton, 1989; Rajan and Cline, 1998). The role of NMDA receptors appears to be specific to this glutamate-gated channel and not to its participation in overall neural activity since enhancing NMDA receptor function by increasing the levels of co-agonist D-serine without affecting glutamatergic neurotransmission alters synaptic maturation through hyperstabilization of axon branches in the developing frog visual system (Van Horn et al., 2017). Similarly, GABAergic depolarizing signals during rodent embryonic cortical neuron development recruit L-type Ca^2+^ channels to regulate neuritogenesis (Maric et al., 2001). Moreover, eliminating this depolarizing action of GABA in a subset of rat ventricular progenitors and the cortical neurons derived from them impairs their morphological maturation (Cancedda et al., 2007).

Neurotransmitter modulation of neuronal differentiation through direct or indirect regulation of ion channel activity is a function shared by neurotrophic factors. Neurotrophin 3 signaling regulates the specification of neuronal phenotype through a voltage-gated Ca^2+^ channel-dependent mechanism that results in a higher number of calbindin-expressing mouse hippocampal pyramidal neurons when Neurotrophin-3 signaling is enhanced and lower when it is decreased (Boukhaddaoui et al., 2001). Brain-derived neurotrophic factor regulates axonal pathfinding during neuronal differentiation by eliciting transient increases in [Ca^2+^]_i_ in growth cones of rat cerebellar granule cells and *Xenopus* spinal cord neurons through the recruitment of TRPC channel activity (Li et al., 2005; Shim et al., 2005; Wang and Poo, 2005).

Ion channels can be mechanically gated and participate in the differentiation of developing neurons. Piezo 1, a mechanosensitive channel, mediates axonal growth and pathfinding of *Xenopus* retinal ganglion cells that direct their growth towards softer tissue (Koser et al., 2016).

Voltage-gated Na^+^ channels are involved in regulating axonal outgrowth and morphology. In zebrafish, knockdown of the Na_v_1.6a alters axonal outgrowth and morphology of dorsally and ventrally projecting secondary motor neurons (Pineda and Ribera, 2008).

Downstream of channel activity local and whole-cell effectors are recruited to change, for example, directionality and growth rate of neurites and neurotransmitter specification, respectively. Ca^2+^ transients recruit activity-dependent transcription factors like CREB (Belgacem and Borodinsky, 2015) or cJUN (Marek et al., 2010) to regulate neurotransmitter phenotype expression in spinal cord neurons. L-type Ca^2+^ channels are recruited downstream of the clustering of the neural cell adhesion molecule 2, inducing submembrane Ca^2+^ spikes that in turn activate the protein tyrosine kinase c-Src, which results in CaMKII activation and increases in filopodia density, neurite outgrowth and branching (Sheng et al., 2015).

Alternatively, ion channels may trigger downstream signaling relevant for neuronal differentiation independently of ion permeation. For example, in some instances, the channel itself regulates transcription, as shown for the C-terminal fragment of Ca_v_1.2 that translocates to the nucleus and acts as a transcription factor controlling rat neuronal differentiation (Gomez-Ospina et al., 2006). Remarkably, a point mutation in this channel that causes Timothy Syndrome, a neurodevelopmental disorder within the autism spectrum disorders, alters dendrite dynamics independent of Ca^2+^ permeation and is dependent on localized RhoA activation (Krey et al., 2013). This mutation appears to interfere with a developmental switch in alternative splicing of this channel, leading to persistent expression of gain of function mutant channels that result in a Ca^2+^-dependent imbalance in the numbers of subtypes of differentiated cortical neurons (Panagiotakos et al., 2019).

## Conclusions

The studies presented demonstrate that ion channels are expressed from the very first stages of neural development. Furthermore, the signaling mechanisms triggered by these ion channels participate in all the relevant cellular processes of early development including neural cell proliferation and neuronal differentiation, mostly through imprinting specific spatiotemporal Ca^2+^ dynamics in developing neural cells. The participation of neural activity *via* ion channel expression throughout neural development poses the question of whether this makes the developing nervous system more vulnerable to “hijacking” of the necessary signaling mechanisms by exogenous unwanted factors. For instance, we have shown that incubating embryos with the AED valproic acid interferes with Ca^2+^ dynamics in neural plate cells and results in NTDs (Sequerra et al., 2018). The counterpart to this apparent vulnerability resides in the plasticity that having neural activity intertwined as a driving developmental mechanism confers upon the nervous system, allowing it to adapt to the changing environment. Indeed, we discovered that animals grown at cold temperatures which exhibit a higher number of motor neurons through a cold temperature-mediated, Ca^2+^ activity-dependent mechanism operating in embryonic spinal cord neurons, exhibit an enhanced escape locomotor performance compared to siblings grown at warm temperatures when tested at cold temperatures (Spencer et al., 2019). Expression of ion channels amplifies the robustness of the developing nervous system by enabling a functional proofreading of the system resulting from the instruction of other developmental cues, according to the surrounding environment.

## Author Contributions

RG, KS and LB wrote the manuscript.

## Conflict of Interest

The authors declare that the research was conducted in the absence of any commercial or financial relationships that could be construed as a potential conflict of interest.
